# Use of a Deep-Learning Algorithm to Guide Novices in Performing Focused Assessment With Sonography in Trauma

**DOI:** 10.1001/jamanetworkopen.2023.5102

**Published:** 2023-03-28

**Authors:** I-Min Chiu, Chun-Hung Richard Lin, Fei-Fei Flora Yau, Fu-Jen Cheng, Hsiu-Yung Pan, Xin-Hong Lin, Chi-Yung Cheng

**Affiliations:** 1 Department of Computer Science and Engineering, National Sun Yat-sen University, Kaohsiung, Taiwan; 2 Department of Emergency Medicine, Kaohsiung Chang Gung Memorial Hospital, Chang Gung University College of Medicine, Kaohsiung, Taiwan

## Abstract

This quality improvement study compares the diagnostic quality and completion time between ultrasonography operators guided by artificial intelligence vs those without such assistance.

## Introduction

Focused Assessment with Sonography in Trauma (FAST) could play a role in reduced time to operative care, shortened hospital length of stay, and lower medical costs.^1^ In a 2005 study, using prehospital FAST was associated with changes to the admitting hospital in 22% of patients.^2,3^ Ultrasonography is highly operator-dependent, and the FAST examination is considered to be among the hardest protocols for image interpretation.^4^ Thus, this study aimed to examine the association between artificial intelligence (AI) guidance and quality of FAST performed by novice ultrasonography operators.

## Methods

This quality improvement study was conducted from March 20 to April 20, 2022. Thirty operators, including 10 registered nurses, 10 nurse practitioners (NPs), and 10 emergency medical technicians (EMTs) without prior experience in performing ultrasonography, were recruited and randomized to a study group: with AI guidance or without AI guidance. The Chang Gung Medical Foundation Institutional Review Board approved the study. Written informed consent was obtained from all participants before the abdominal ultrasonography. We followed the SQUIRE reporting guideline.

To enable AI guidance, we integrated the deep learning (DL)–based guidance algorithm that we developed^5^ into an OpenCV application that captures images from ultrasonography machines and provides real-time quality feedback (Video). This DL algorithm was found to classify qualified and nonqualified images with an accuracy of 0.941.^5^ Each operator was instructed to perform FAST examination over the Morrison pouch in 10 healthy patients (the model group) in the same order to obtain a 5-second clip of the standard view within 3 minutes.

**Video.** Demonstration of Real-Time Artificial Intelligence–Guided Ultrasonography With Quality and Result Feedback The image quality and confidence level of the interpretation are displayed in the upper left corner, and the interpretation result of the presence of ascites is displayed in the upper right corner. When the scanning position is correct, a prompt will appear to instruct the operator to “hold the probe.”

The primary outcome was diagnostic quality, which our panel of 3 expert echocardiographers (F.-J.C., H.-Y.P., X.-H.L.) assessed independently. Diagnostic quality was rated from 1 to 5, with higher scores representing better quality (eAppendix in Supplement 1). A rating of 4 or higher was considered to be acceptable quality for clinical use. The secondary outcome was time to complete examinations. We performed regression analysis to adjust for confounding factors (Table) and analyze the association of AI guidance with diagnostic quality.

**Table.** Regression Analysis of AI Guidance on Different Study Outcomes

Outcome	Variable	OR (95% CI)	*P* value
Acceptable quality score ≥4	Work tenure, y	1.04 (0.95 to 1.12)	.31
	Model group BMI	0.96 (0.85 to 1.08)	.51
	With AI guidance	3.82 (1.78 to 8.20)	<.001
Diagnostic quality score	Work tenure, y	0.00 (−0.03 to 0.03)	.82
	Model group BMI	−0.05 (−0.09 to 0.00)	.06
	With AI guidance	0.40 (0.12 to 0.68)	.005
Time to complete examination, s	Work tenure, y	−0.66 (−2.00 to 0.68)	.33
	Model group BMI	2.37 (0.28 to 4.45)	.03
	With AI guidance	14.36 (2.03 to 26.71)	.02

Abbreviations: AI, artificial intelligence; BMI, body mass index (calculated as weight in kilograms divided by height in meters squared); OR, odds ratio.

Two-sided *P* < .05 indicated statistical significance. Data analysis was performed with SPSS, version 26 (IBM).

## Results

A total of 300 ultrasonography scans were created by 30 operators. The model group consisted of 5 males and 5 females, with a median (IQR) age of 46 (35-50) years and body mass index (calculated as weight in kilograms divided by height in meters squared) ranging from 21.5 to 27.8. The intraclass correlation coefficient for diagnostic quality scores was 0.97 (95% CI, 0.96-0.98), indicating excellent reliability.

The median (IQR) quality score (5 [4-5] vs 4 [3-5]; *P* = .02) and the mean (SD) rate of acceptable quality score (126 [84%] vs 102 [68%]; *P* = .002) were higher in operators with AI guidance vs those without AI guidance. Additionally, AI guidance was associated with a higher quality score (odds ratio [OR], 0.40; 95% CI, 0.12-0.68; *P* = .005) and rate of acceptable quality (OR, 3.82; 95% CI, 1.78-8.20; *P* < .001) (Table).

Furthermore, AI guidance was associated with longer examination time (OR, 14.36; 95% CI, 2.03-26.71 seconds; *P* = .02) (Table), which was mostly observed in the first few rounds of operations (Figure). In a subgroup analysis of operators’ occupational position, AI guidance was associated with better diagnostic quality among NPs and EMTs (Figure).

**Figure.**
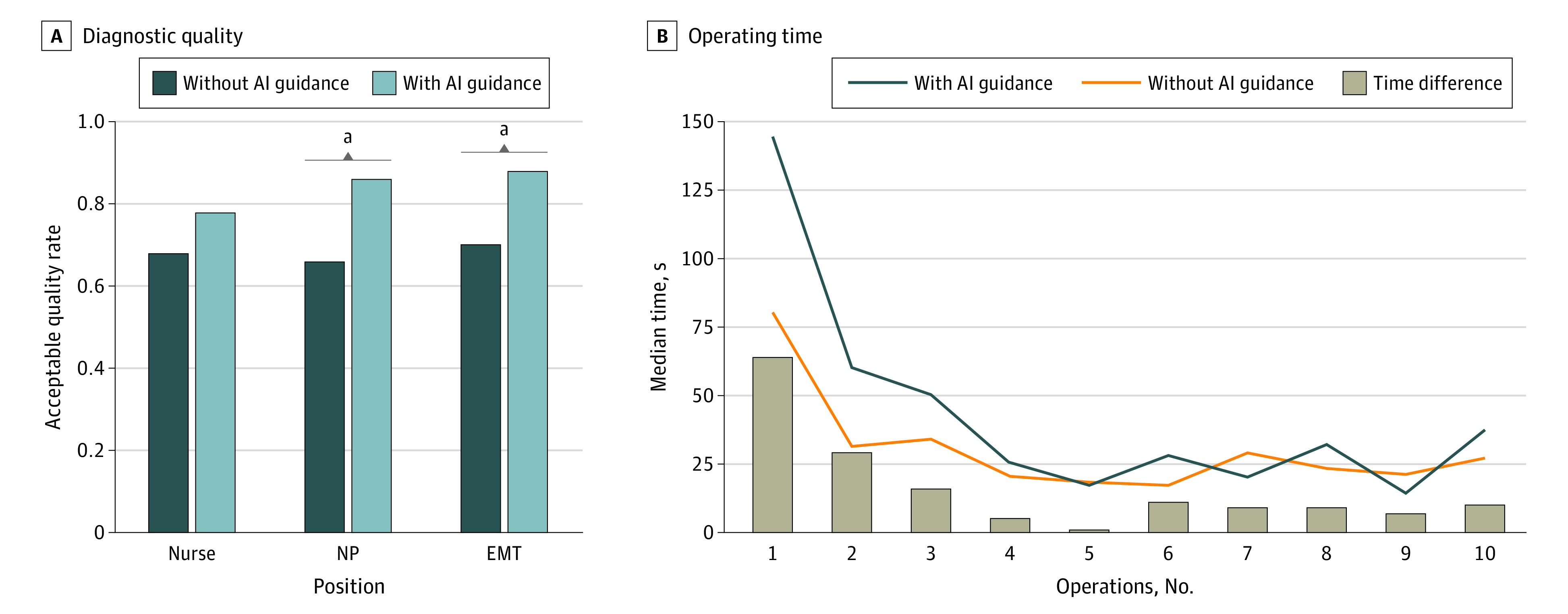
Comparison of Diagnostic Quality and Operating Time Between Operators With Artificial Intelligence (AI) Guidance and Without AI Guidance EMT indicates emergency medical technician; NP, nurse practitioner. ^a^Statistical difference in the acceptable quality rate.

## Discussion

This study showed that the DL algorithm can guide novices to obtain satisfactory diagnostic images over the Morison pouch. The diagnostic quality score and the rate of acceptable clips were significantly higher with AI guidance. Although initially it may take longer to complete an examination with AI guidance, it is expected that the learning curve will be lower for novices practicing FAST. A study limitation is that it was conducted in a laboratory rather than a clinical trauma setting. To fully evaluate the potential benefits of using AI in the management of patients with traumatic injury, further research in clinical deployment is necessary.

## Notes

Supplement 1. **eAppendix.** Grading Scale for Quality of Ultrasound Scan

Supplement 2.
Data Sharing Statement
