# Supplementary material for: Use of a Deep-Learning Algorithm to Guide Novices in Performing Focused Assessment With Sonography in Trauma
Source: JAMA Netw Open. 2023 Mar 28;6(3):e235102. doi: 10.1001/jamanetworkopen.2023.5102 (PMC10051044; doi:10.1001/jamanetworkopen.2023.5102)
Supplement: Supplement 1. — eAppendix. Grading Scale for Quality of Ultrasound Scan [file jamanetwopen-e235102-s001.pdf]

Supplementary Online Content

Chiu IM, Lin CHR, Yau FFF, et al. Use of a deep-learning algorithm to guide novices in performing Focused Assessment With Sonography in Trauma. *JAMA Netw Open*. 2023;6(3):e235102. doi:10.1001/jamanetworkopen.2023.5102

eAppendix. Grading Scale for Quality of Ultrasound Scan

This supplementary material has been provided by the authors to give readers additional information about their work.

© 2023 Chiu IM et al. *JAMA Network Open.*

eAppendix. Grading Scale for Quality of Ultrasound Scan

points	description
**1**	Neither liver nor kidney identified
**2**	Part of the liver or kidney identified
**3**	Both liver and kidneys were seen, but only a small part of them
**4**	Most of the liver and kidneys were seen, but can be better
**5**	Great view of Morison pouch

© 2023 Chiu IM et al. *JAMA Network Open.*
