# Supplementary material for: Use of a Deep-Learning Algorithm to Guide Novices in Performing Focused Assessment With Sonography in Trauma
Source: JAMA Netw Open. 2023 Mar 28;6(3):e235102. doi: 10.1001/jamanetworkopen.2023.5102 (PMC10051044; doi:10.1001/jamanetworkopen.2023.5102)
Supplement: Supplement 2. — Data Sharing Statement [file jamanetwopen-e235102-s002.pdf]

Data Sharing Statement

Chiu. Use of a Deep-Learning Algorithm to Guide Novices in Performing Focused Assessment With Sonography in Trauma. *JAMA Netw Open*. Published March 28, 2023. doi:10.1001/jamanetworkopen.2023.5102

Data
Data available: Yes
Data types: Deidentified participant data
How to access data: The datasets used and/or analyzed during the current study are available from the corresponding author upon reasonable request.
When available: With publication

Supporting Documents
Document types: None

Additional Information
Who can access the data: researchers whose proposed use of the data has been approved
Types of analyses: researchers whose proposed use of the data has been approved
Mechanisms of data availability: with a signed data access agreement
